# Parameter Optimization, Morphological and Histological Characteristics of Accurate Bone Ablation by Femtosecond Laser: An In Vitro Study

**DOI:** 10.3390/bioengineering12030217

**Published:** 2025-02-21

**Authors:** Yiyang Wang, Shanshan Liang, Yongsheng Zhou, Fusong Yuan, Hongqiang Ye

**Affiliations:** 1 Department of Prosthodontics, Peking University School and Hospital of Stomatology, National Center of Stomatology, National Clinical Research Center for Oral Diseases, National Engineering Research Center of Oral Biomaterials and Digital Medical Devices, Beijing Key Laboratory of Digital Stomatology, Beijing 100081, China; wyiyang0116@163.com (Y.W.); kqzhouysh@hsc.pku.edu.cn (Y.Z.); 2 Center of Digital Dentistry, Department of Prosthodontics, Second Clinical Division, Peking University School and Hospital of Stomatology, National Center of Stomatology, National Clinical Research Center for Oral Diseases, National Engineering Research Center of Oral Biomaterials and Digital Medical Devices, Beijing Key Laboratory of Digital Stomatology, Research Center of Engineering and Technology for Computerized Dentistry Ministry of Health, Beijing 100081, China; dentist_lss@163.com; 3 Center of Digital Dentistry, Department of Prosthodontics, Peking University School and Hospital of Stomatology, National Center of Stomatology, National Clinical Research Center for Oral Diseases, National Engineering Research Center of Oral Biomaterials and Digital Medical Devices, Beijing Key Laboratory of Digital Stomatology, Research Center of Engineering and Technology for Computerized Dentistry Ministry of Health, Beijing 100081, China

**Keywords:** ultrafast laser, femtosecond laser, bone ablation, surgical robot, parameter optimization, laser energy density

## Abstract

The use of femtosecond laser for bone ablation has been demonstrated in numerous studies; however, the clinical application requires further optimization to meet safety, accuracy, and efficiency standards. This study aims to optimize the energy density parameter of a robot-controlled femtosecond laser surgical system for bone ablation by assessing temperature changes, ablation efficiency, and ablation effects. Furthermore, the morphological and histological characteristics of bone tissue were compared with those of conventional mechanical methods. The results indicated that a laser energy density of 1.05 J/cm^2^ was optimal for bone ablation, maintaining the bone surface temperature below 47 °C and achieving an ablation efficiency of 0.145 mm^3^/s. The deviations in cavity diameters were significantly smaller for the laser group (6.58 ± 18.09 μm) compared to the bur group (80.09 ± 45.45 μm, *p* < 0.001, *N* = 5 per group). Femtosecond laser ablation produced cleaner cavity margins with minimal bone debris accumulation. Additionally, the adjacent Volkmann and Haversian canals retained their normal morphology, indicating limited mechanical and thermal damage to the bone tissue. The robot-controlled femtosecond laser system demonstrated the potential for achieving safe, accurate, efficient, and clean bone ablation, offering promising prospects for clinical applications.

## 1. Introduction

Bone surgeries, including orthognathic surgery, bone tumor resection, joint replacement, and dental implantation, demand surgical instruments that minimize trauma, reduce operation time, and promote wound healing [1,2]. However, conventional tools such as drills, saws, and chisels often generate friction and vibration, causing mechanical and thermal damage to bone tissues [3]. Additionally, bone and metal debris may increase the risk of infection and delay healing [4,5]. Despite advancements such as piezosurgery, which offers improved accuracy [6,7] and tissue selectivity [8,9,10], these tools remain inefficient for handling dense cortical bones and large-scale bone tissues [8,11]. This underscores the need for innovative technologies that address these limitations.

The advent of laser technology has introduced new approaches to bone surgery by enabling non-contact, precise, and minimally invasive ablation [12]. Among various lasers, the Er:YAG laser has demonstrated particular efficacy due to its wavelength (2940 nm) being close to the water absorption peak [13], allowing for the photothermal evaporation of mineralized tissues [14,15,16,17]. However, limitations such as micro-explosions caused by rapid water evaporation [18,19,20] and the potential for thermomechanical damage [21,22,23] have restricted the clinical application of conventional laser technology.

To address the limitations of conventional laser technology, femtosecond laser has been extensively studied in recent years and has found widespread applications in the medical field [24,25]. Femtosecond laser, with the pulse width measured in femtoseconds (1 fs = 10^−15^ s), can be focused on an extremely small area, achieving ablation accuracy at the micron or even sub-micron level [26,27,28]. When focused on tissues, the femtosecond laser can rapidly ionize the target with minimal heat accumulation [29,30]. Lo et al. [31] reported that femtosecond laser ablation, compared to mechanical drilling, minimized collateral damage and promoted faster bone healing. While these properties have made femtosecond lasers a promising tool for bone tissue surgeries, achieving a balance between ablation efficiency and biosafety remains a challenge in clinical applications [32,33,34,35].

Previous studies on femtosecond laser parameters for bone ablation (Table 1) have shown significant variability in terms of bone specimens, laser systems, and experimental settings, and the reported parameters have yet to meet clinical requirements. Therefore, further optimization of laser parameters is necessary. A robot-controlled femtosecond laser surgical system was developed and suitable parameters, such as repetition rate, scanning speed, and scanning path for bone ablation, were preliminarily explored in previous works [36,37]. Based on this, the present study focused on laser energy density, which is of utmost concern in clinical settings. This study aims to optimize the energy density parameter of a robot-controlled femtosecond laser surgical system for bone ablation by assessing temperature changes, ablation efficiency, and ablation effects; the morphological and histological characteristics of bone tissue were compared with those of conventional mechanical methods.

## 2. Materials and Methods

### 2.1. Preparation of Sheep Tibiae Specimens

Fresh sheep tibiae were collected from a local abattoir. Ten tibiae segments were prepared, each approximately 5 cm in length. After removing the skin, muscle, and other soft tissues, the bones were thoroughly cleaned with saline. The bone surfaces were sequentially polished using 800-, 1000-, and 2000-grit wet sandpapers to ensure uniform smoothness. Each segment was then fixed onto a silicone impression material (PERFIT^®^, HUGE, Shanghai, China) base with the polished surfaces aligned parallel to the base. All cavity preparations were completed within 2 h after specimen preparation.

### 2.2. Cavity Preparation by Femtosecond Laser and Temperature Monitoring

**Figure 1:**
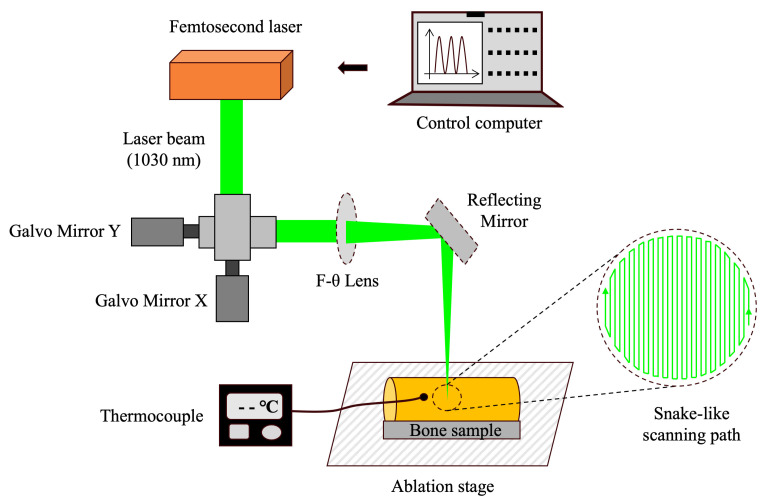
Schematic of the robot-controlled femtosecond laser surgical system.

The schematic of the robot-controlled femtosecond laser surgical system is illustrated in Figure 1. The system consists of a femtosecond laser (Tangerine, Amplitude System, Pessac, France), mirrors, lenses, a movable 3D platform, a mini robot, and a robotic control system [36,46]. The laser operated at a wavelength of 1030 nm, with a repetition rate of 100 kHz and a scanning speed of 1900 mm/s. Four laser energy densities were set, which were 0.58 J/cm^2^, 0.81 J/cm^2^, 1.05 J/cm^2^, and 1.28 J/cm^2^, respectively.

The ablation platform was adjusted to ensure that the upper surface of the bone specimens was aligned with the laser focus. A snake-like scanning path with a total ablation time of 80 s was preprogrammed into the control system. Under robotic control, cavities with a diameter of 4 mm were prepared with the energy density group Set 1–4 (10 per group). Before cavity preparation, 1.5 mm diameter holes were drilled 0.5 mm from the planned cavity margins to insert thermocouple sensors (309/K, Center, Taiwan, China). The positions of these holes were precisely adjusted using the galvanometer system. During the laser ablation process, the thermocouple sensors continuously monitored and recorded the surface temperature of the bone specimens.

### 2.3. Comparison of Ablation Efficiency and Surface Roughness Among Four Laser Sets

Ablation efficiency (*η*), defined as the volume of bone tissue removed by the laser per second, was evaluated for each specimen (*N* = 10 per group) using a three-dimensional (3D) laser scanning microscope (VK-X200, Keyence Corporation, Osaka, Japan). The depth of each cavity was measured with the integrated 3D measurement software (VK Analyzer 3.5.0.40, Osaka, Japan). For each cavity, three random areas were selected and measurements were conducted three times per area to ensure accuracy. Ablation efficiency was calculated using the following formulas:

*η* = *V/t* (1)

*V* = *πd*^2^*h*/4 (2)

*η* represents laser ablation efficiency, *V* represents the volume of bone tissue removed, *t* represents the time of laser ablation, *d* represents diameter of the cavity, and *h* represents the depth of the cavity.

Arithmetic Mean Roughness (Ra) was determined as the average of the absolute deviations of the surface profile from a reference line within a specified measurement area [47]. The 3D laser scanning microscope acquired (X, Y, Z) data of the sample surface to generate a topography map. For each cavity (*N* = 10 per group), three random areas were selected and Ra was measured across five 100 μm × 100 μm grids within each area. A reference plane was generated by the measurement software and height deviations from this plane were averaged to obtain the Ra value.

### 2.4. Comparison of Morphology and Elemental Composition Among Four Laser Sets

The bone specimens (*N* = 10 per group) were placed on the stage of a stereomicroscope (SMZ25, Nikon, Tokyo, Japan). The illumination intensity and focus were adjusted to clearly visualize the top view of each cavity. Images were captured using a high-resolution camera attached to the microscope and all files were saved in TIFF format.

Following stereomicroscope imaging, the specimens were sequentially dehydrated in 70% ethanol for 24 h, then 90% ethanol for 1 h, and, finally, 100% ethanol for 1 h. After conductive coating, the cavities were examined using a Field Emission Scanning Electron Microscope (SU8010, Hitachi, Tokyo, Japan). Magnification and focus settings were optimized to obtain clear images of the ablated bone surfaces. Elemental composition, specifically the mass percentages of C, O, Ca, and P, was analyzed using Energy-Dispersive Spectroscopy (EDS). For each cavity, three equally sized areas were randomly selected for analysis.

### 2.5. Morphological Comparison Between Laser and Bur Group

Cavities with a diameter of 4 mm and a depth of 2 mm were prepared (laser group, *N* = 10) using the optimized femtosecond laser parameters, following the method described above. Identical cavities (bur group, *N* = 10) were also created using a conventional mechanical method with a dental implant machine (CHIROPRO 3rd Gen, Bien Air, Lausanne, Switzerland) and burs (Straumann, Straumann Group, Basel, Switzerland).

All specimens were initially photographed under a stereomicroscope (SMZ25, Nikon, Tokyo, Japan). Subsequently, half of the specimens (*N* = 5 per group) were dehydrated and examined using Scanning Electron Microscopy (SEM). The diameter of each cavity was measured three times, and the deviation was calculated as the difference between the measured diameter and the preset diameter. The remaining specimens (*N* = 5 per group) were decalcified in 10% Ethylenediaminetetraacetic acid (EDTA) for histological sectioning.

### 2.6. Histological Comparison Between Laser and Bur Group

After decalcification, the specimens (*N* = 5 per group) were sequentially dehydrated in 50%, 70%, 95%, and 100% ethanol, each for 1 h. The dehydrated specimens were then cleared in xylene for 30 min and embedded in paraffin. Sections were cut to a thickness of 7 µm. Following deparaffinization, the sections were subjected to Hematoxylin–Eosin (HE) and Masson’s Trichrome staining. After staining, the sections were dehydrated through graded alcohols, cleared in xylene, and sealed with a mounting medium for microscopic observation.

### 2.7. Statistical Analysis

Statistical analyses were performed using SPSS 22.0 software (IBM Corporation, Armonk, NY, USA). The sample size of this study referred to similar studies [33,36]. For the study of laser parameter optimization, cavities with a diameter of 4 mm were prepared with a simple size of *N* = 10 for each laser setting (Set 1-4). These samples were utilized for stereomicroscopic observation, 3D laser scanning microscopy, Ra measurement, SEM observation, and EDS analysis. In the study comparing the microstructure and histological characteristics of bone tissue between the laser bur group, a sample size of *N* = 10 was used for both the laser group and the bur group. Among these, *N* = 5 per group were allocated for SEM observation and *N* = 5 per group were allocated for histological sections. Each experiment and measurement were repeated at least three times to ensure reliability. Quantitative data, which conformed to normal distribution and exhibited equal variance, were presented as mean ± standard deviation (mean ± SD). An independent samples *t*-test was employed to compare cavity diameters between the laser and bur groups. One-way ANOVA was used to compare bone surface temperature, ablation efficiency, surface roughness, and elemental mass percentages among the different laser settings. When significant differences were detected, Tukey’s Honest Significant Difference (HSD) post hoc test was conducted for pairwise comparisons. *p* < 0.05 was considered statistically significant. All statistical graphs were generated using Prism 10.0 (GraphPad Software, San Diego, CA, USA).

## 3. Results

### 3.1. The Temperature Changes of Bone Surface During Laser Ablation

**Figure 2:**
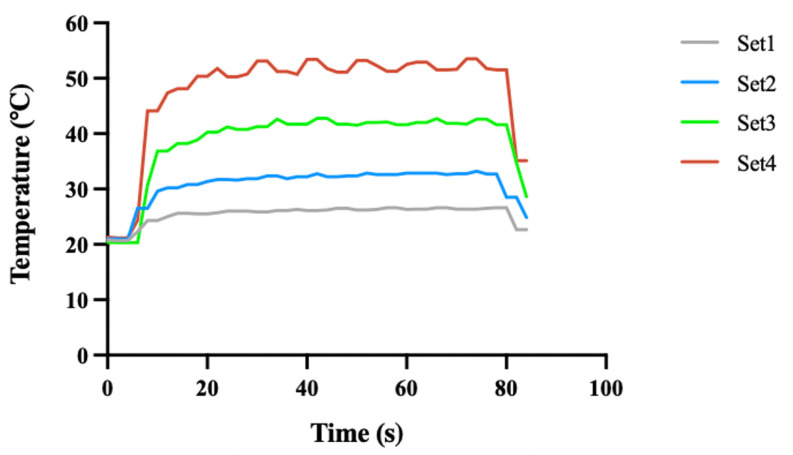
Temperature of bone surface during laser ablation. Set 1, 2, 3, and 4 correspond to laser energy densities of 0.58 J/cm^2^, 0.81 J/cm^2^, 1.05 J/cm^2^, and 1.28 J/cm^2^, respectively. The ablation time for each laser set was 80 s.

Figure 2 illustrates the changes in bone surface temperature recorded by a thermocouple during 80 s femtosecond laser ablation time under four energy density sets. At the onset of laser ablation, the bone surface temperature gradually increased, reaching a peak level where it fluctuated slightly before gradually returning to room temperature after the ablation ended. The higher the laser energy density, the faster the initial rise in bone surface temperature and the higher the average temperature during ablation. As shown in Table 2, the maximum bone surface temperatures during the laser ablation process were 26.6 °C, 33.2 °C, 42.8 °C, and 53.5 °C for laser set 1–4, respectively.

### 3.2. Comparison of Ablation Efficiency and Surface Roughness Among Four Laser Sets

**Figure 3:**
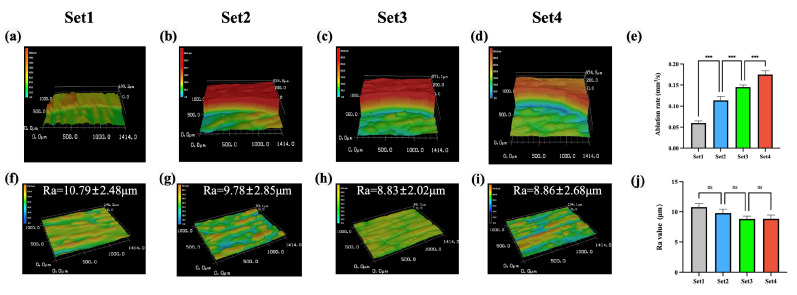
Comparison of ablation efficiencies and surface roughness among four laser sets. (**a**–**d**) Three-dimensional images of the margin of the cavities. (**e**) Comparation of ablation efficiency. (**f**–**i**) Three-dimensional surface morphology images. (**j**) Comparison of the surface Ra values (***: *p* < 0.001, ns: *p* > 0.05, *N* = 10 per group).

Figure 3a–d illustrate the 3D images of the margin of the cavities, and Figure 3e indicates that, as laser energy density increases, the bone ablation efficiency improves (*p* < 0.001, *N* = 10 per group). For the ablation time of 80 s, the average depths of the cylindrical cavities with a diameter of 4 mm, prepared by laser set 1–4, were 0.380 mm, 0.723 mm, 0.924 mm, and 0.111 mm, respectively. The corresponding average bone ablation efficiencies were 0.060 mm^3^/s, 0.114 mm^3^/s, 0.145 mm^3^/s, and 0.175 mm^3^/s, respectively.

Figure 3f–i demonstrate that the mean Ra values of the ablated bone surfaces were 10.79 μm, 9.78 μm, 8.83 μm, and 8.86 μm for laser set 1–4, respectively. The differences between these groups were not statistically significant (*p* > 0.05, *N* = 10 per group).

### 3.3. Comparison of Morphology and Elemental Composition Among Four Laser Sets

**Figure 4:**
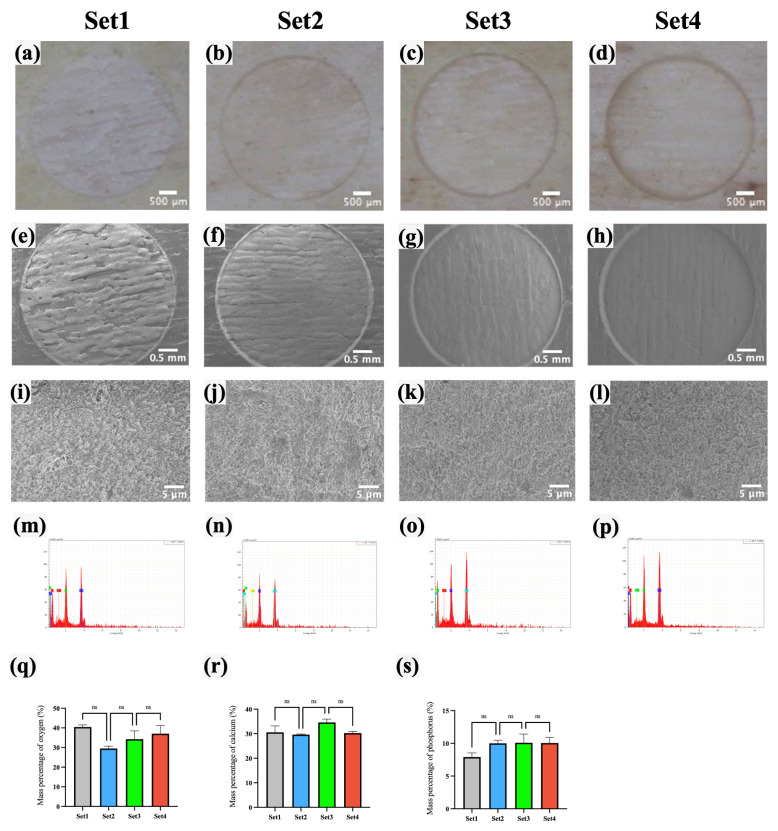
Morphology and elemental composition analysis after ablating by four laser sets. (**a**–**d**) Stereomicroscopy images. (**e**–**l**) SEM images. (**m**–**p**) EDS spectra. (**q**–**s**) Comparison of the surface elemental mass percentages of O, Ca, and P. (ns: *p* > 0.05, *N* = 10 per group.).

As shown in Figure 4e–l, the bone surfaces ablated with the four laser sets exhibited a uniformly rough and porous structure, with no visible cracks resulting from the laser ablation. To assess the impact of femtosecond laser ablation on the chemical properties of bone tissue, the elemental mass percentages of oxygen (O), calcium (Ca), and phosphorus (P) on the ablated bone surfaces were compared across the four laser sets. As shown in Figure 4q–s and Table 3, the average mass percentages of O for laser set 1–4 were 40.46%, 29.51%, 34.29%, and 37.08%, respectively; for Ca, they were 30.58%, 29.64%, 33.29%, and 30.25%; and, for P, they were 6.57%, 8.33%, 10.42%, and 9.39%. No significant differences were observed between these groups (*p* > 0.05, *N* = 10 per group). Figure 4m–p further indicate that carbon (C) was not detected on the bone surfaces by EDS analysis after ablation with any of the four laser sets.

### 3.4. Morphological Comparison Between Laser and Bur Group

**Figure 5:**
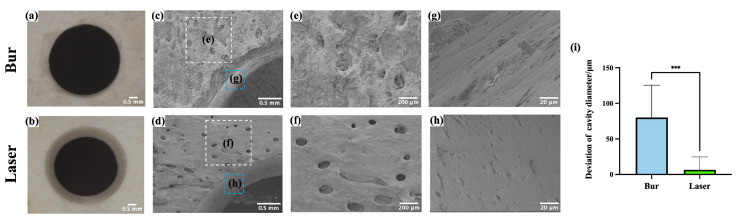
Microstructure of bone surface after laser ablation and mechanical drilling. (**a**,**b**) Representative images of cavities under stereomicroscopy. (**c**,**d**) Images of the margin of cavities. (**e**,**f**) Magnified images of the white boxed areas in (**c**,**d**,**g**,**h**). Magnified images of the blue boxed areas in (**c**,**d**). (**i**) Comparison of cavity diameter deviation prepared by laser and mechanical burs. (***: *p* < 0.001, *N* = 5 per group.).

Figure 5a,b show that the margins of cavities prepared by femtosecond laser were neater and clearer compared to those prepared by mechanical drilling. In Figure 5c,d, it is evident that the cavities prepared by mechanical drilling were surrounded by noticeable bone debris, whereas those created by the femtosecond laser were cleaner and free of significant bone debris. Figure 5c–f present images of Volkmann canals near the margin of the cavities at different magnifications. Mechanical drilling generated substantial bone debris that blocked the Volkmann canals, while the canals near the margins of the cavities ablated by femtosecond laser maintained the normal morphology with clear openings. Figure 5g,h show that the lateral walls of cavities prepared by mechanical drilling were rough and exhibited burs; moreover, the openings of Haversian canals were blocked by bone debris and the normal morphology was destroyed. In contrast, laser ablation resulted in clean, smooth lateral walls with clearly visible Haversian canal openings.

The diameters of the cavities prepared by mechanical drilling and laser ablation were 4080.09 ± 45.45 μm and 4006.58 ± 18.09 μm, respectively. As shown in Figure 5i, the deviations in cavity diameters were 80.09 ± 45.45 μm for the bur group and 6.58 ± 18.09 μm for the laser group. The difference was statistically significant (*p* < 0.001, *N* = 5 per group). The accuracy of laser ablation was superior to that of mechanical drilling, this may be attributed to the vibrations during drilling and wear of the burs.

### 3.5. Histological Comparison Between Laser and Bur Group

**Figure 6:**
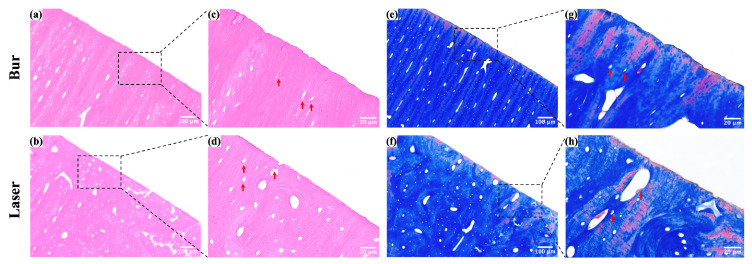
Histological images of the margin of cavities after laser ablation and mechanical drilling. (**a**,**b**) HE staining images of the margin of cavities. (**c**,**d**) Magnified images of the boxed areas in (**a**,**b**). (**e**,**f**) Masson staining images of the margin of cavities. (**g**,**h**) Magnified images of the boxed areas in (**e**,**f**).

Histological images of the cavity margins after laser ablation and mechanical drilling are shown in Figure 6 (*N* = 5 per group). The red arrows indicate the lacunae with viable osteocytes; no obvious thermal damage was observed in either the laser or bur groups. However, compared to the serrated, rough, and irregular edges created by mechanical drilling, the edges of the cavities produced by laser ablation were neat and uniform.

## 4. Discussion

This study optimized the energy density parameter of the robot-controlled femtosecond laser surgical system through in vitro experiments. It also compared the ablation effects between the femtosecond laser and conventional mechanical method in terms of accuracy, bone surface morphology, and histological characteristics after ablation.

A femtosecond laser energy density of 1.05 J/cm^2^, which meets clinical requirements for safety and efficiency, was selected as the optimal parameter. The corresponding average bone ablation efficiency was 0.145 mm^3^/s.

When the energy density was up to 1.28 J/cm^2^, the maximum temperature of the bone surface during laser ablation exceeded 50 °C. Studies have shown that maintaining the bone surface temperature at 47 °C for more than 60 s can cause irreversible damage to osteocytes, and temperatures above 50 °C may lead to the denaturation of alkaline phosphatase, thereby adversely affecting the healing and self-organization of the bone defect [18,33,48]. Therefore, to ensure safety, laser set 4 is not suitable for bone ablation.

The results of previous studies suggested that femtosecond laser ablation efficiency for bone tissue ranges from 0.8 × 10^−4^ mm^3^/s to 0.99 mm^3^/s (Table 1). These variations are influenced by factors such as the femtosecond laser wavelength, repetition rate, energy density, scanning speed, cooling method, and the type of bone tissue. For instance, Zhang et al. [32] reported femtosecond laser ablation efficiency of 0.99 mm^3^/s and 0.82 mm^3^/s on sheepshank with an energy density of 14.15 J/cm^2^ under air-cooling and water-cooling conditions, respectively. Similarly, Gemini et al. [34] achieved an ablation efficiency of 0.66 mm^3^/s on pig femur using a 515 nm femtosecond laser with an average power of 6.27 W under air cooling. These studies reported higher bone ablation efficiencies than the present study, without causing significant thermal damage to the tissue. This may be primarily due to the cooling methods used in these studies. Research has shown that, while air cooling and water cooling can effectively reduce temperature and remove some bone debris [32], they may cause plasma plume displacement when airflow interacts with the plasma during laser ablation, leading to uneven energy distribution of the laser beam. Additionally, the air and water flow may cause scattering and absorption of the laser beam, further negatively impacting ablation outcome [49,50]. Therefore, for accuracy and patient comfort in clinical application, no cooling methods were used during laser ablation in this study.

The surface morphology and chemical composition are critical indicators of the thermal damage during femtosecond laser ablation [42]. In this study, the bone surfaces ablated using the four laser parameters showed no visible carbonization. The microstructure and elemental composition of O, Ca, and P on the bone surface exhibited no significant differences. This is likely because the laser parameters chosen for this study were based on prior studies, resulting in a relatively narrow range of laser energy densities. Zhang et al. [32] found that the ratios of Ca/P and (Ca + Mg)/P on the surface of bone tissue without thermal damage after laser ablation were similar to that of normal bone tissue. However, these ratios increased on the surface of bone tissue with thermal damage, potentially due to the reduction of organic components and the evaporation of water caused by elevated temperatures; thus, P and Mg that are firmly bound to water molecules are reduced. In the study by Gemini et al. [34], cracks appeared on the surface of thermally damaged bone tissue after laser ablation, resulting in increased surface roughness. They explained that C increased in the early stage of thermal damage due to tissue carbonization but, as organic components were consumed, the proportion of inorganic components such as Ca in the bone tissue increased.

The preservation of the normal structure and function of Volkmann canals and Haversian canals, which accommodate and interconnect blood vessels and nerves, is crucial for bone tissue repair and regeneration [51,52]. Additionally, studies have indicated that, if bone debris generated during mechanical drilling is not completely removed, it may clog the drill hole [3], thereby increasing drilling force torque [26,53] and bone surface temperature [54].

The accuracy of laser ablation was superior to that of mechanical drilling; this may be attributed to the vibrations during drilling and wear of the burs. The results suggest that laser ablation provides significant advantages for bone surgeries with high accuracy requirements. Studies have indicated that severe heat accumulation during laser ablation can damage bone tissue, leading to the formation of acellular voids [32] and even tissue carbonization [28,32], which appeared more deeply stained in histological sections [33]. Therefore, the histological results once again confirmed that the laser set 3 selected for this study ensured acceptable biological safety and achieved higher-quality bone tissue ablation compared to the conventional mechanical drilling method. However, in vitro bone tissue lacks the blood supply and metabolic processes that are present in vivo, resulting in inevitable differences in its response compared to bone in living organisms. Nonetheless, it can still provide insights into the extent of thermal and mechanical damage to the tissue [32,33].

The limitations of this study include the exclusive use of in vitro sheep tibiae, which may not fully represent the complexity and heterogeneity of human bone structures. Additionally, in vitro experiments do not account for physiological factors present in living organisms, such as blood flow, immune responses, and bone regeneration processes. This research solely evaluated the implant machine and specific burs used for dental implantation, thereby not addressing the variability of mechanical methods employed in clinical applications. In future studies, we plan to assess the long-term effects of femtosecond laser ablation through in vivo experiments, thereby providing more reliable data to support the clinical application of femtosecond lasers in bone surgery.

## 5. Conclusions

This study optimized the energy density parameter of the robot-controlled femtosecond laser surgical system through in vitro experiments and compared its ablation performance with conventional mechanical methods in terms of accuracy, bone surface morphology, and histological characteristics. The main conclusions of this study are as follows:

- The optimized femtosecond laser energy density in this study met the requirements of safety, efficiency, and accuracy for clinical applications.
- The robot-controlled femtosecond laser system enabled automatic, safe, accurate, efficient, and clean ablation of bone tissue, demonstrating significant potential for clinical applications in bone surgery.

## Figures and Tables

**Table 1:** Summary of femtosecond laser parameters for bone tissue ablation.

Author, Year	Bone Type	Wavelength	Repetitive Rate	Pulse Width	Scan Rate	Energy Density	Ablation Threshold (Pulse)	Ablation Rate
***In vitro * studies**								
Liang et al., 2022 [36]	Rabbit femur	1030 nm	200 kHz	500 fs	4000 mm/s	NA	NA	36 μm/ 5 scans
Gemini et al., 2021 [34]	Porcine femur	515 nm	250 kHz	350 fs	4000 mm/s	NA	NA	0.66 mm^3^/s
Zhang et al., 2020 [32]	Sheepshank	1030 nm	200 kHz	230 fs	2000 mm/s	14.15 J/cm^2^	NA	0.99 mm^3^/s
Rico et al., 2019 [38]	NA	1030 nm	60 kHz	240 fs	100 mm/s	6.1 J/cm2	NA	0.053 mm^3^/s
Su et al., 2014 [30]	Bovine articular cartilage	1700 nm	5 kHz	NA	NA	NA	NA	0.8 × 10^−4^ mm^3^/s
Emigh et al., 2012 [39]	Porcine scapulae	800 nm	1 kHz	170 fs	NA	NA	1.75 J/cm^2^ (1000)	NA
Cangueiro et al., 2012 [40]	Bovine femur	1030 nm	10 Hz	500 fs	NA	NA	0.79 J/cm^2^ (1)	NA
Lim et al., 2009 [41]	Bovine femur	775 nm	3 kHz	150 fs	NA	NA	2.70 J/cm^2^ (1)	NA
McCaughey et al., 2009 [29]	Otic capsule	1053 nm	10 kHz	700 fs	NA	NA	8.5 J/cm^2^ (1)	NA
Liu et al., 2007 [42]	Porcine femur	1030 nm	10 kHz	Below 900 fs	NA	NA	NA	0.02 mm^3^/s
Girard et al., 2007 [28]	Porcine mandible	775 nm	1 kHz	200 fs	NA	NA	0.69 J/cm^2^ (1000)	NA
Wieger et al., 2007 [18]	Bovine spongiosa, compacta, and cartilage	1040 nm	1 kHz	330 fs	NA	NA	0.54–0.82 J/cm^2^ (1)	NA
Armstrong et al., 2002 [43]	Human incus and stapes	1053 nm	10 Hz	350 fs	NA	2.0 J/cm^2^	NA	1.26 mm/pulse
***In vivo* studies**								
Lo et al., 2012 [31]	Mouse calvaria	800 nm	1 kHz	150 fs	4 mm/s	NA	NA	NA
Cloutier et al., 2010 [44]	Mouse calcaria	775 nm	1 kHz	<200 fs	NA	8.0 J/cm^2^	NA	NA
Girard et al., 2007 [45]	Mouse calcaria	775 nm	1 kHz	<200 fs	200 mm/s	NA	NA	NA

NA: not available. Inclusion Criteria: (1) Studies on femtosecond laser ablation of bone tissue; (2) Studies with specific description of the laser parameters; (3) Studies implrmented on bone tissue; (4) Both in vitro and in vivo studies; (5) Original research articles; (6) Published in English. Exclusion Criteria: (1) Studies not involving femtosecond laser but focusing on other lasers (e.g., nanosecond or picosecond lasers); (2) Studies not targeting bone tissue; (3) Studies without detailed description on femtosecond laser parameter; (4) Non-English publications; (5) Review articles, case reports, case series.

**Table 2:** Maximum temperature and temperature increase during laser ablation.

Laser Set	Energy Density (J/cm^2^)	Maximum Temperature (°C)	Maximum Temperature Increase (°C)
Set 1	0.58	26.4 ± 0.2	5.8 ± 0.2
Set 2	0.81	33.1 ± 0.1	12.2 ± 0.1
Set 3	1.05	42.7 ± 0.1	22.4 ± 0.1
Set 4	1.28	53.2 ± 0.3	32.1 ± 0.3

**Table 3:** Elemental mass percentage of bone surface after laser ablation.

Laser Set	Elemental Mass Percentage (%)		
	O	Ca	P
Set 1	40.46 ± 1.84	30.58 ± 4.51	6.57 ± 1.24
Set 2	29.51 ± 2.09	29.64 ± 0.37	8.33 ± 2.20
Set 3	34.29 ± 7.28	33.29 ± 6.50	10.42 ± 3.67
Set 4	37.08 ± 7.09	30.25 ± 1.27	9.39 ± 2.67

## Data Availability

The original contributions presented in this study are included in the article. Further inquiries can be directed to the corresponding authors.
